# Methods for Estimating Personal Disease Risk and Phylogenetic Diversity of Hematopoietic Stem Cells

**DOI:** 10.1093/molbev/msad279

**Published:** 2023-12-20

**Authors:** Jack M Craig, Glenn S Gerhard, Sudip Sharma, Anastasia Yankovskiy, Sayaka Miura, Sudhir Kumar

**Affiliations:** Institute for Genomics and Evolutionary Medicine, Temple University, Philadelphia, PA, USA; Department of Biology, Temple University, Philadelphia, PA, USA; Department of Medical Genetics and Molecular Biochemistry, Lewis Katz School of Medicine, Temple University, Philadelphia, PA, USA; Institute for Genomics and Evolutionary Medicine, Temple University, Philadelphia, PA, USA; Department of Biology, Temple University, Philadelphia, PA, USA; Institute for Genomics and Evolutionary Medicine, Temple University, Philadelphia, PA, USA; Department of Biology, Temple University, Philadelphia, PA, USA; Institute for Genomics and Evolutionary Medicine, Temple University, Philadelphia, PA, USA; Department of Biology, Temple University, Philadelphia, PA, USA; Institute for Genomics and Evolutionary Medicine, Temple University, Philadelphia, PA, USA; Department of Biology, Temple University, Philadelphia, PA, USA

**Keywords:** biological age, hematopoietic stem cells, somatic mutations, cancer risk

## Abstract

An individual's chronological age does not always correspond to the health of different tissues in their body, especially in cases of disease. Therefore, estimating and contrasting the physiological age of tissues with an individual's chronological age may be a useful tool to diagnose disease and its progression. In this study, we present novel metrics to quantify the loss of phylogenetic diversity in hematopoietic stem cells (HSCs), which are precursors to most blood cell types and are associated with many blood-related diseases. These metrics showed an excellent correspondence with an age-related increase in blood cancer incidence, enabling a model to estimate the phylogeny-derived age (phyloAge) of HSCs present in an individual. The HSC phyloAge was generally older than the chronological age of patients suffering from myeloproliferative neoplasms (MPNs). We present a model that relates excess HSC aging with increased MPN risk. It predicted an over 200 times greater risk based on the HSC phylogenies of the youngest MPN patients analyzed. Our new metrics are designed to be robust to sampling biases and do not rely on prior knowledge of driver mutations or physiological assessments. Consequently, they complement conventional biomarker-based methods to estimate physiological age and disease risk.

## Introduction

Somatic aging is characterized by an unrelenting accumulation of genetic variants that become a mutational burden with potentially significant health consequences, particularly in tissues with high cellular turnover (Fancello et al. 2019; Sha et al. 2020). Hematopoietic stem cells (HSCs) bearing newly evolved variants can increase in numbers due to proliferative and/or survival advantage, leading to clonal expansions. Such clonally expanded HSCs can result in clonal hematopoiesis of indeterminate potential (CHIP) in circulating blood cells. CHIP increases with age, paralleling the increased risk of hematological neoplasms and cardiovascular disease (Bick et al. 2020; Nachun et al. 2021; Younes et al. 2023). This pattern is often associated with driver mutations in some genetic loci, such as DNMT3A and TET2 (Buscarlet et al. 2017; Bailey et al. 2018).

Many studies identify CHIPs by the presence of such driver mutations, with their reported prevalence rates ranging from ∼1% in young people (<40 yr old) to >15% in those over 65 (Groarke and Young 2019; Bick et al. 2020; Fabre et al. 2022). These drivers emerge from a background of somatic mosaicism that develops continuously over time (Groarke and Young 2019; Bick et al. 2020; Fabre et al. 2022). However, few studies have focused on using putative passenger (nondriver) variation to assess disease risk. Advancements in single-cell sequencing of HSC genomes now provide base-resolution profiles of all genetic changes in somatic cells (Lee-Six and Kent 2020; Van Egeren et al. 2021; Fabre et al. 2022; Mitchell et al. 2022), offering new avenues for developing quantitative models to complement the analysis of driver mutations.

**Fig. 1.**
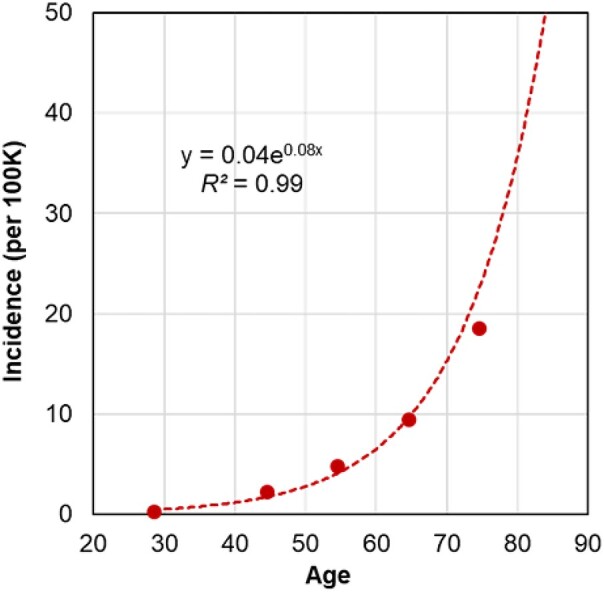
The incidence of blood cancer by age per 100K individuals. The incidence of MPN increases exponentially by age according to the following function: incidence (per 100K) = 0.04×e^0.08×Age^. Rates of incidence were obtained from Hultcrantz et al. (2020). This pattern mirrors the known incidence rates of other blood cancers such as leukemia and myeloma (Cancer Research UK 2016 to 2018, International Classification of Diseases [ICD] codes ICD-10 C91 to C95 and 2016 to 2018, ICD-10 C90, respectively).

In this study, we measured the accumulation of single nucleotide alterations (SNAs) in somatic genomes of HSCs and assessed temporal changes in HSC phylogenetic diversity with age. We present novel approaches to measure the decay of phylogenetic diversity of HSC genomes generated from single-cell sequencing data. We explored the relationships between the number of SNAs and the decay of phylogenetic diversity with the age-related incidence of cancer in populations (Fig. 1). Based on the observed patterns, we developed a model to estimate HSC phylogeny–derived age (phyloAge) to predict the increased risk of disease. We applied these measures to HSC phylogenies of individuals suffering from myeloproliferative neoplasms (MPNs) to estimate increased cancer risk independent of commonly used panels of driver mutations or other biomarkers.

## Results and Discussion

### Accumulation of Genetic Variation in HSCs in the Healthy

We utilized the most comprehensive single-cell sequencing data publicly available for HSCs (Mitchell et al. 2022). This consists of whole-genome sequences of DNA obtained from 3,579 colonies of cells derived from single immunophenotypic HSCs (Lin−CD34+CD38−CD45RA−) that were sorted using flow cytometry and then cultured to produce single-cell–derived hematopoietic colonies from 10 healthy individuals spanning the human lifespan.

**Fig. 2.**
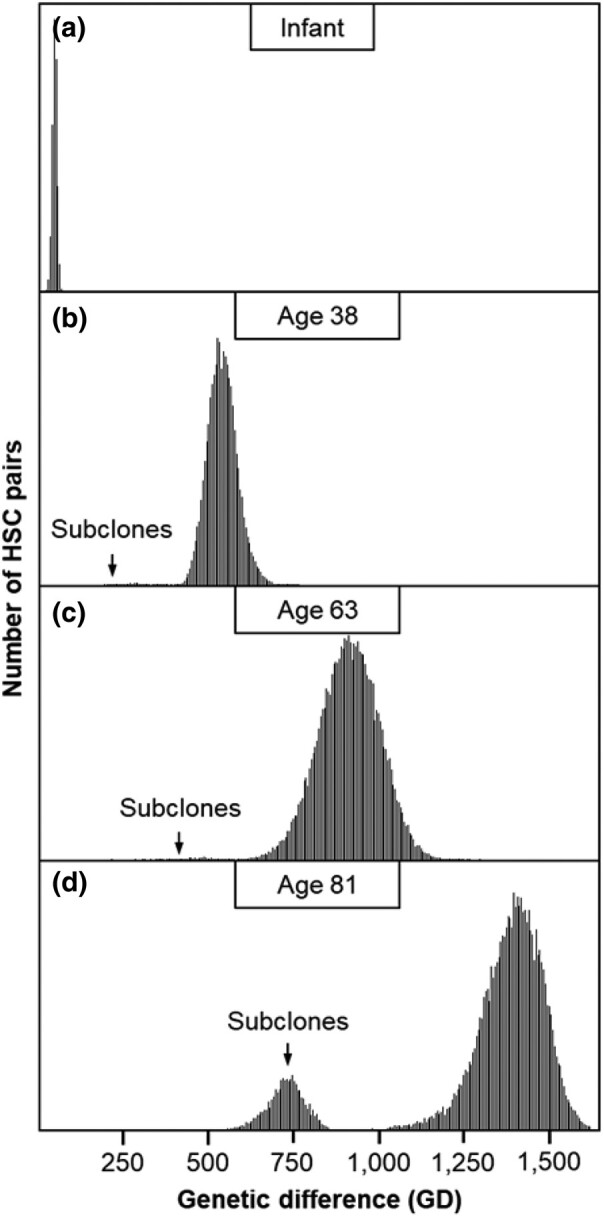
Distributions of GDs between HSCs. Distributions are shown for a) an infant, b) a 38-yr-old, c) a 63-yr-old, and d) an 81-yr-old. Subclones arising from later in life are marked by an arrow, which forms a tail or a distribution to the left of the primary peak corresponding to the HSCs that arose during embryogenesis.

We calculated the number of genetic differences (GDs) between HSC sequences for each individual. We divided these by 2 to generate per-lineage estimates for each pair of HSCs. The distribution of these GDs was unimodal in infants, consistent with the rapid initial HSC expansion during embryogenesis, which results in limited HSC lineage divergence and thus similar GDs (Fig. 2a). With increasing age, the GDs increase due to the steady accumulation of new variants, resulting in longer GDs in 38-yr-olds and 63-yr-olds (Fig. 2b and c). These adult distributions are characterized by long tails of shorter GDs, representing the gradual accumulation of more recently diverged subclonal HSCs that are more closely related to one another than to any of the founder HSCs. In an 81-yr-old individual, these developed into a secondary peak reflecting the increasing prevalence of subclonal HSCs (Fig. 2d), consistent with CHIPs that arise after the establishment of the initial population of HSC clones during embryogenesis and become more prominent with age (Groarke and Young 2019; Ayachi et al. 2020; Fabre et al. 2022).

### The Tempo of Sequence Variation Accumulation in HSCs

**Fig. 3.**
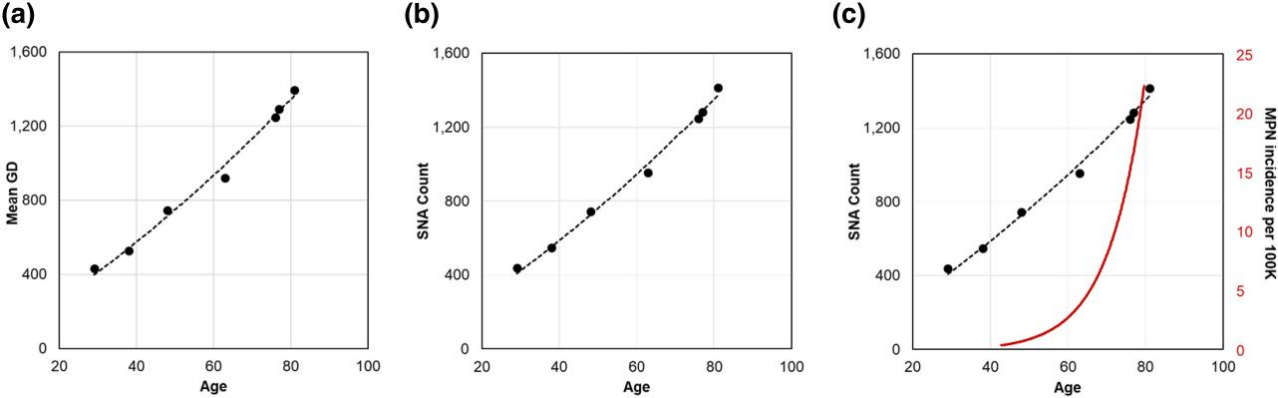
Accumulation of sequence variation over time in HSCs. Relationship of a) GDs and b) SNA counts with age for 7 healthy adult individuals. These relationships are curvilinear, as a second-degree polynomial fits the data better than a linear regression in both cases (*P* < 0.01). c) Tempos of SNA and GD increases do not explain the much more rapid increase in the age-related incidence of MPN.

Figure 3a shows the relationship between the central values of primary peaks in GD distributions and the ages of 7 healthy adults. The relationship is nearly linear, but the null hypothesis of a linear fit was rejected at *P* < 0.01 when compared with a second-order polynomial fit. The curvilinear relationship was also found for SNAs, which were detected directly by comparing HSC genomes with the germline genome (*P* < 0.01; Fig. 3b). These findings differ from previous reports of a constant molecular clock for HSC evolution (Osorio et al. 2018; Brown et al. 2019; Dietlein et al. 2020; Mitchell et al. 2022; Williams et al. 2022). Importantly, the curvilinear relationship between age and SNA counts and GDs does not explain the exponential increase in the age-related incidence of blood cancers (Fig. 3c).

### Decay of Phylogenetic Diversity with Age

**Fig. 4.**
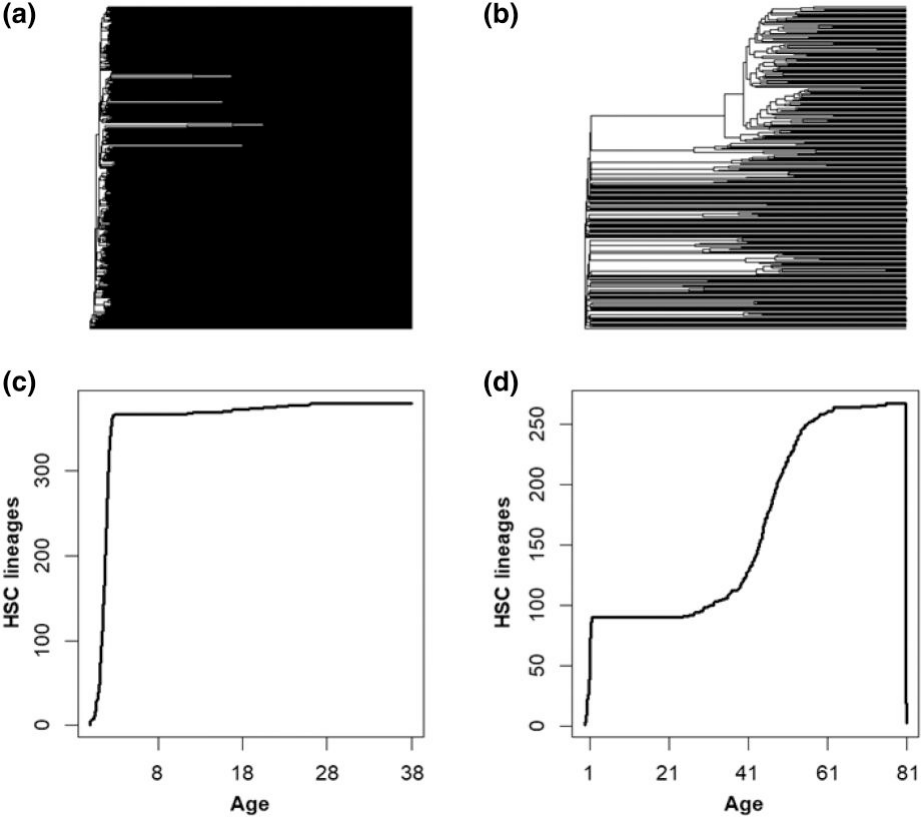
The HSC phylogenies of 2 healthy individuals and changes in their phylogenetic biodiversity. a) The HSC phylogeny of a 38-yr-old healthy individual, which exhibits <10 HSC lineage divergences after the embryonic phase. b) The HSC phylogeny of an 81-yr-old healthy individual, which has many large clades containing subclonal HSCs. Only one-third of the tips are direct descendants of the embryonic HSCs. c) The LTT plot from the 38-yr-old exhibits an initial rapid diversification in HSCs, followed by a period of growth, with minimal increase in total lineages thereafter. d) The LTT plot from an 81-yr-old healthy individual exhibits an initial rapid diversification in HSCs, a period of growth, and then a second period of increase later in life.

To seek an explanation (or at least a better correlation) for the exponential age-related increase in cancer risk, we explored phylogenies reconstructed using SNAs in HSC genomes as an alternative method for characterizing patterns of age-related change in HSC. An individual's total number of HSC lineages has been shown to be established during embryogenesis and remains relatively stable throughout life (Lee-Six et al. 2018; Jaiswal and Ebert 2019; Ayachi et al. 2020; Mitchell et al. 2022). As expected then, the HSC phylogeny of a 38-yr individual exhibits extensive polyclonality, visible as many early-branching lineages, with only rare instances of evolutionary bifurcations (Fig. 4a). In contrast, the HSC phylogeny of an elderly individual (81 yr old) has many expanding lineages (CHIPs) with many descendant subclones (Fig. 4b). In this case, subclonal HSCs comprise over one-third of all HSCs, which means that subclonal HSCs that originated after birth appear to replace ancestral HSC lineages.

Since the ancestral HSCs diversified early and evolved independently of others, they represent a greater phylogenetic diversity than the more recently diverged subclonal lineages that share a genetic history with closely related subclonal HSCs. Therefore, the displacement of ancestral HSC lineages by subclonal HSCs represents a decay in phylogenetic diversity with age (Mitchell et al. 2021). The lineages-through-time (LTT) plots reveal these phenomenological trends in biodiversity loss based on the temporal distribution of branch points in the phylogenetic trees. In the LTT plot of the 38-yr-old (Fig. 4c), the total number of HSC lineages is established early and remains similar over time. In contrast, the LTT plot of the elderly individual shows an additional phase of HSC diversification and the loss of phylogenetic diversity (Fig. 4d).

### New Phylogenetic Measures of Biodiversity Decay

**Fig. 5.**
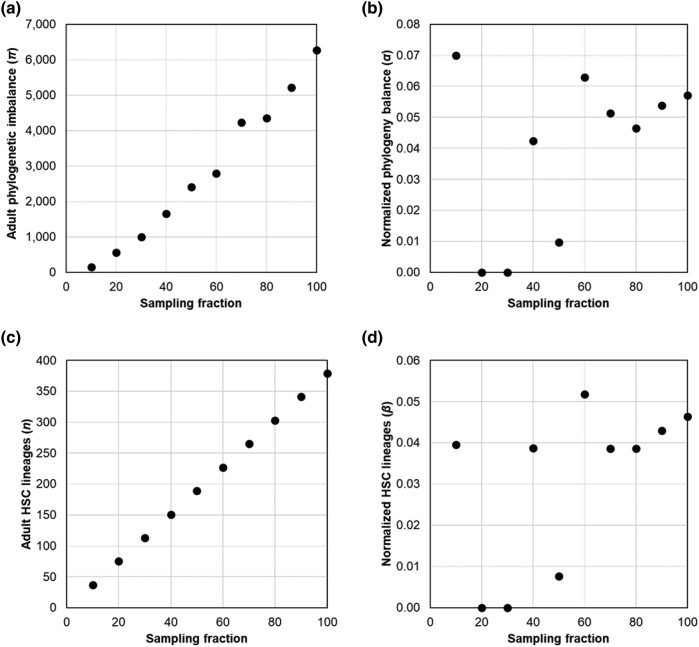
Normalized metrics account for lineage sampling. a) The relationship of the imbalance of adult HSC phylogenies (*π_a_*) with data richness (*R*^2^ = 0.98) and other info. b) The standardized phylogeny imbalance (*α*) shows a low correlation with data richness (*R*^2^ = 0.16; *P* > 0.05). c) The relationship of the number of tips present in the adult HSC phylogeny (*n*) is the same as the sampling fraction (*R*^2^ = 1). d) The standardized tip count (*β*) is also not highly correlated with data richness (*R*^2^ = 0.16; *P* > 0.05).

We explored the use of 2 biodiversity metrics to quantify the decline in phylogenetic biodiversity with age in single-cell HSC phylogenies: HSC phylogeny imbalance (*π*) and the number of HSC lineages (*n*). Phylogeny imbalance (Colless 1982) is the sum of absolute differences in the sizes of the descendant clades for every internal node in the tree. Computationally, the absolute size difference between the 2 descendant clades of a node is calculated for every node, and these values are summed for all the internal nodes in the phylogeny to obtain the imbalance metric. Due to the CHIP events, the imbalance grows in the HSC phylogeny with age. However, *π* and *n* depend on the number of HSCs sampled, as shown in Fig. 5a and c, respectively. In these analyses, we randomly removed subsets of HSC lineages from the HSC phylogeny of a 38-yr-old healthy individual (KX002) at 10% intervals, resulting in a set of 10 phylogenies ranging from 100% to 10% of the total lineages sampled. The probability of a given lineage appearing in a phylogeny is called “sampling fraction,” which may be treated as a measure of data richness in the HSC phylogeny. We then calculated phylogenetic imbalance and lineage counts for each. The imbalance of HSC phylogenies (*π*) scaled linearly with data richness (*R*^2^ = 0.98; Fig. 5a) as did the number of tips (*n*) present in the HSC phylogeny (*R*^2^ = 1; Fig. 5c).

So, we developed 2 novel metrics (*α* and *β*) to quantify the decline in phylogenetic biodiversity with age in single-cell HSC phylogenies. These metrics are designed to be robust to the number of HSCs sampled. The first metric (*α*) measures the standardized change in *π* over an individual's lifetime after birth as the natural logarithm of the ratio of contemporary and ancestral values of *π*:


(1) $$\alpha =\log_{2}\;(\pi /\pi_{a})=\log_{2}\;(\pi )-\log_{2}\;(\pi_{a})$$

The contemporary value of *π* is estimated using the whole HSC phylogeny. The ancestral imbalance (*π_a_*) is obtained by cropping the phylogeny at a time point equivalent to the inflection point in its LTT plot, where the initial HSC lineage diversification ends at or near birth (see *Materials and Methods*). The *α* metric has a minimum value of 0 at birth because *π* and *π_a_* will be equal. A base-2 logarithm is used because phylogenetic branching is typically a doubling process. HSC phylogenies’ *α* estimates were not significantly correlated with the number of HSCs sampled (*R*^2^ = 0.16; *P* > 0.05), unlike the estimate of *π* (compare Fig. 5a and b).

The second metric (*β*), based on the number of HSC clonal lineages (*n*), is also intrinsically normalized. It is the logarithm of the ratio of observed lineages (*n*) and inferred ancestral lineages that retained (*n_a_*):


(2) $$\beta =\log_{2}\;(n/n_{a})=\log_{2}\;(n)-\log_{2}\;(n_{a})$$

The *β* metric is 0 for young, healthy individuals who have not lost any ancestral HSC lineages. However, it increases over time as expanding subclones displace the ancestral HSCs, reducing overall diversity. We find no significant relationship between *β* and data richness (*R*^2^ = 0.16; *P* > 0.05 and *R*^2^ = 0.16; *P* > 0.05; Fig. 5c and d).

### Relationship of *α* and *β* with Age-Related Increase in Blood Cancer

**Fig. 6.**
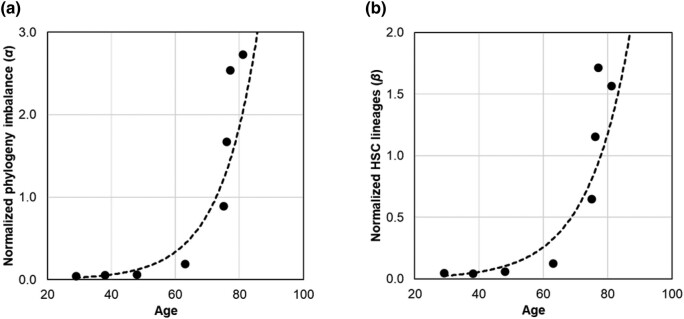
The relationship of phylogenetic diversity decay metrics with age and age-related cancer risk. a) Normalized phylogeny imbalance (*α*) in HSC phylogenies from healthy individuals increases following an exponential function: *α* = 0.002×e^0.09×age^ (*R*^2^ = 0.89). b) Normalized HSC lineage count (*β*) increases following an exponential function: *β* = 0.003×e^0.08×age^ (*R*^2^ = 0.87).

We estimated *α* and *β* metrics using HSC phylogenies of 8 healthy adults. Both exhibit an exponential relationship with age (Fig. 6). The diversity decay increases exponentially, remaining small until about 65 yr and increasing rapidly thereafter. These patterns were highly concordant with that observed for the cancer risk, with the correlation coefficients between the MPN risk and the biodiversity metrics of *R*^2^ = 0.89 (*P* < 0.01) for *α* and *R*^2^ = 0.87 for *β* (*P* < 0.01).

### Estimating PhyloAge of HSC Phylogenies

The relationships of *α* and *β* estimates with the age of healthy adults and their cancer risk prompted us to develop a model to estimate what we call HSC phyloAge. In this case, both *α* and *β* were used as phylogenetic markers analogous to epigenetic markers of age (Hannum et al. 2013; Horvath 2013; Simpson and Chandra 2021). We optimized the values of parameters using a numerical Gauss–Newton algorithm, aiming to minimize the sum of squared differences between the observed and the predicted age (see *Materials and Methods*).

**Fig. 7.**
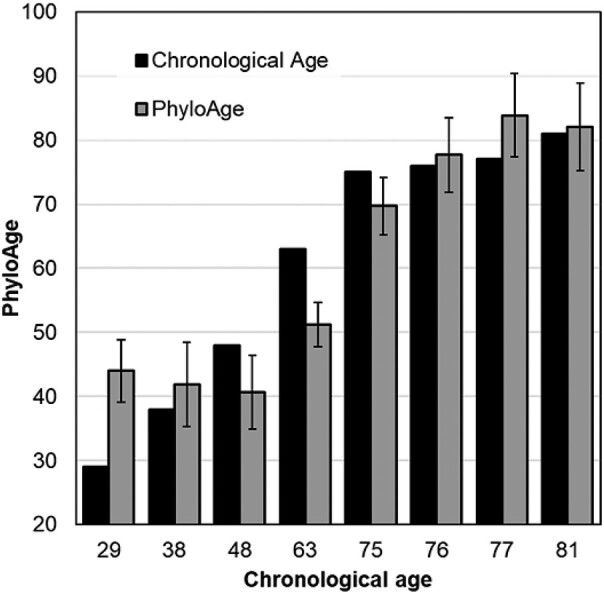
The relationship of the estimated phyloAge with the chronological age of healthy adults. We estimated phyloAges using the composite model incorporating *α* and *β*, with 95% confidence intervals derived from the LOO analysis.

In leave-one-out (LOO) analyses, the phyloAge model predictions achieved a correlation coefficient of 0.82 with the chronological age. The absolute difference between phyloAge and chronological age, referred to as the residualAge, was 6.6 yr on average (Fig. 7). The predictions for younger individuals, where *α* and *β* do not change much over time, had higher residualAge (3.8 to 15.0 yr). The residualAge was <3.7 yr for individuals 65 yr and older. We also explored the use of Shannon's biodiversity index (Whittaker 1972; Mitchell et al. 2022) but found that its inclusion in the phyloAge model did not improve the accuracy of predictions.

The performance of the phyloAge model was comparable with that reported for epigenetic clocks in some recent studies. For example, GrimAge2 was trained using thousands of methylation profiles to predict chronological age and showed a correlation of 0.78 to 0.95 between true and predicted ages (Lu et al. 2019, 2022), whereas the correlation was 0.94 for phyloAges. DeepMAge, a deep learning method trained on 4,930 methylation profiles (Galkin et al. 2021), was reported to predict age with a median error of 2.8 yr, which is better than phyloAges for young people but similar for older people. These results suggest that phyloAge could yield results comparable with existing methods, with the possibility of improvement when single-cell HSC-sequencing data become available for additional healthy adults.

### Estimates of PhyloAges for the HSC Phylogenies of MPN Patients

**Fig. 8.**
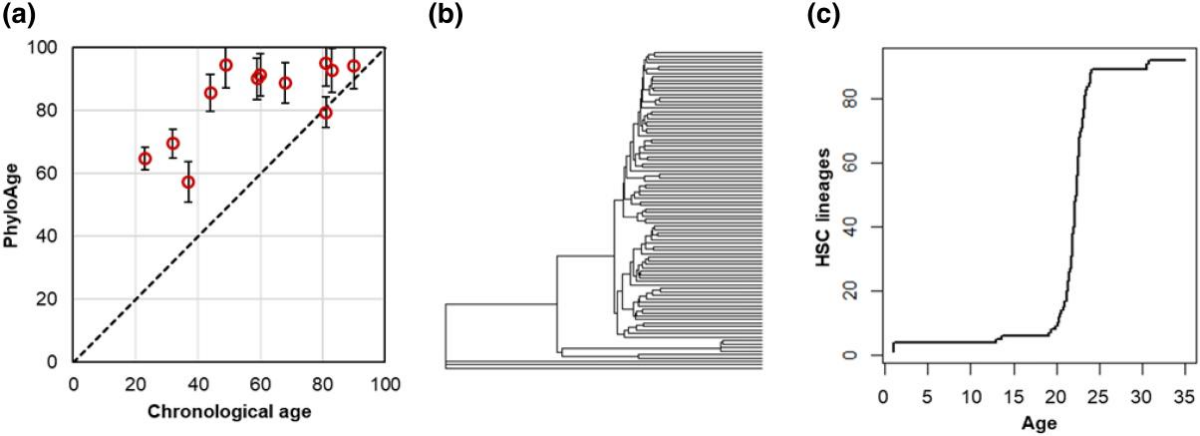
Application of the phyloAge model to the phylogenies of MPN patients. a) PhyloAges of individuals with MPN are substantially greater than their true chronological ages. A 1:1 relationship (dashed line) is shown for comparison. b) The HSC phylogeny of a 49-yr-old individual with MPN. Only 3 of 92 HSCs trace their direct origin back to the root, and one subclonal lineage gives rise to a vast majority of HSCs present at age 49, which displayed almost all the primary HSC lineages. c) The logged LTT plot from a 49-yr-old individual with MPN exhibits an initial rapid diversification in HSCs, a period of growth, and then a second period of increase later in life, much like the older healthy person, but much sooner.

Next, we applied our metrics and methods to the HSC phylogenies of 12 MPN patients aged 23 to 83 yr (Williams et al. 2022). These data were acquired similarly to those of healthy individuals (Mitchell et al. 2022). The MPN phyloAge estimates were consistently older than the chronological ages (Fig. 8a). For example, a 49-yr-old MPN patient received a phyloAge of 94.4 yr, indicating that their HSC biodiversity had decayed by an additional 45.4 yr beyond their chronological age. This is evident upon comparing their HSC phylogeny (Fig. 8b) with that of the oldest healthy person (81 yr old; Fig. 4b). In fact, the MPN patient has lost all but 3 ancestral HSC lineages, which represents a greater loss in genetic diversity and a greater change in phylogeny shape than that of a healthy person almost 4 times their age (Fig. 4b). In general, young MPN patients’ LTTs, and even cellular phylogenies, resemble those of much older healthy individuals. That is, MPN is associated with HSC diversity loss earlier in life due to the dominance of subclonal HSC lineages. Overall, residualAges discriminated between the healthy adults and MPN patients until the age of 80, with the residualAges > 33 for MPN patients <80 yr old (Fig. 8a).

We sought to compare the performance of residualAge based on the phyloAge model for MPN patients with those reported for biological ages derived from methylation biomarkers, which have been widely used since their introduction (Horvath 2013). One study that used DNA methylation markers to estimate physiological ages in MPN patients specifically found an average residualAge of just 0.5 yr. However, the residualAge estimates based on DNA methylation vary extensively among cancer types. Weidner et al. (2014) reported small differences for aplastic anemia (average 11.7 yr) and dyskeratosis congenita (average 16.5 yr), whereas Zhu et al. (2019) reported much larger differences for many cancers (up to 50 yr). DeepMAge predicted an average residualAge of 1.7 yr for ovarian cancer. Therefore, residualAge differences can be large for some types of cancers, including MPN examined in this study.

Unlike residualAges of MPN phylogenies, we did not see a significant difference between the counts of cancer-associated driver variants between the healthy individuals and those with MPN, as the average number of drivers in healthy individuals and MPN patients were quite similar (4.5 and 3.9 per individual, respectively) and not significantly different (*P* > 0.75). Therefore, while drivers are frequently implicated in causing CHIPs (Brown et al. 2019; Dietlein et al. 2020), their counts do not discriminate between healthy and diseased individuals. However, we found SBS9 mutational signatures in CHIP lineages of elderly MPN patients only (ages > 80 yr), which showed limited discrimination when using phyloAges. SBS9 is a key cancer-associated mutational signature characterized by T > G mutations and is induced by somatic hypermutation, which is often reported in the lymphoid samples and myeloid cancer cells (Alexandrov et al. 2020; Degasperi et al. 2022). SBS9 was not present in any of the healthy individuals. Therefore, SBS9 may be useful as an additional biomarker for detecting the emergence of MPN in conjunction with phyloAge when the residualAge is small. But, more data are needed to test these suggestions.

### Assessing Increased Cancer Risk Using PhyloAge

We found the residualAge, the difference between the HSC phyloAge and chronological age, to be naturally related to the fold increase in cancer risk due to excess aging, because the reported trends in MPN cancer incidence with age are described by an exponential function (0.04×e^0.08×age^; *R*^2^ = 0.99). In this case, the equation for the fold increase in the risk of MPN:


(3) $$\begin{array}{rl} \mathrm{Fold}\text{-}\mathrm{increased}\;\mathrm{risk} & =\frac{0.04\times e^{0.08\times \mathrm{phyloAge}}}{0.04\times e^{0.08\times \mathrm{chronological}\;\mathrm{age}}}\\ & =e^{0.08\times (\mathrm{phyloAge}-\mathrm{chronological}\;\mathrm{age})} \end{array}$$

This equation shows that the increased risk is a function of the difference between phyloAge and chronological age. It translates premature HSC aging into an estimate of increased cancer risk. Using this framework, it should be possible to develop similar equations for other types of cancers and diseases that are influenced by changes in the HSCs.

Applying the above equation to the HSC phylogeny in MPN patients predicts that a 23-yr-old person, whose phyloAge is 64.76, will have a 34-fold increase in their probability of developing MPN. Similarly, individuals aged 40 to 65 exhibited a 27-fold increase in risk, while those aged 65 to 81 showed a more than 2-fold increase in risk. Based on these results, phyloAge shows promise as a tool for forecasting MPN risk based solely on the phenomenological metrics of phylogenetic diversity decay.

## Conclusion

HSCs give rise to myeloid cells that differentiate into red blood cells, platelets, neutrophils, basophils, monocytes, eosinophils, and lymphoid progenitors that include T and B lymphocytes and natural killer cells (Ogawa 1993; Mikkola and Orkin 2006). CHIP appears to arise from the acquisition of somatic mutations in HSC genomes that permit and/or drive clonal expansion over time to produce an age-dependent increase in blood and immune cell mosaicism (Nachun et al. 2021; Ahmad et al. 2023; Goldman et al. 2023; Singh and Singh 2023). Consequently, CHIP is associated with an increased risk of hematological malignancies and cardiovascular and pulmonary diseases (Jaiswal et al. 2017; Wong et al. 2023).

We have presented 2 novel phylogeny shape metrics (*α* and *β*), based on traditional ecological biodiversity measures, to capture the decay of diversity of HSC phylogenies reconstructed using SNAs. These measures increase exponentially with age and are concordant with the age-related increases in cancer incidence, fulfilling the need for quantitative phenomenological descriptions of the time-dependent structure of HSC phylogenies. We have also presented a method to estimate HSC phyloAge using *α* and *β* that significantly exceeds the chronological age of HSCs in MPN patients (average 59%, up to 182% in extreme cases).

This age difference was found to distinguish between healthy and diseased individuals more effectively than other quantitative descriptions utilizing DNA methylation (Hannum et al. 2013; Horvath 2013; Weidner et al. 2014; Bell et al. 2019; Lu et al. 2019, 2022; Galkin et al. 2021; Seale et al. 2022; Dabrowski et al. 2023). HSC phyloAge was more effective at predicting physiological ages and discerning between healthy people and those with blood cancer than the GD or total number of SNAs accumulated. Further, while few of the somatic SNAs an individual acquires in their lifetime are expected to be cancer-associated driver mutations (Bailey et al. 2018; Brown et al. 2019; Nussinov et al. 2019), even restricting our analysis to these drivers was not sufficient to discern between healthy people and those with cancer, as the counts of drivers in HSC genomes of individuals suffering from MPN were not sufficiently different from those in healthy individuals (Mitchell et al. 2022; Williams et al. 2022). Interestingly, the difference between phyloAge and chronological age forecasts a fold increase in cancer risk. This could find application in clinical and research settings to track the temporal rate of deterioration of HSCs when personal single-cell sequencing at different time points becomes more routine.

As the field of biological aging research continues to evolve, we anticipate the development of new and more useful approaches, such as chronic sterile inflammation (Franceschi et al. 2018), glycomics (Borelli 2014; Krištić et al. 2014), and lipidomics (Beyene et al. 2020; Slade et al. 2021). These tools are already showing a promising ability to identify many serious diseases (Horvath and Ritz 2015; Ding and Rexrode 2020; Miyoshi et al. 2020; Wang et al. 2020; Gaunitz et al. 2021). There are even many new tools being developed to give consumers access to their blood health data (Csordas et al. 2022), though these have not yet been able to bridge the gap between physiological age and disease risk.

Regarding phyloAge, we anticipate that advancements in single-cell sequencing technology and the availability of richer cancer incidence data with age will enable more accurate HSC phylogeny–based evolutionary modeling for age and risk assessment. With an increase in single-cell sequencing data from younger healthy individuals, where changes in genomic diversity are more subtle, we expect to understand the natural trajectory of HSC evolution better. These analyses and predictive models will also characterize aging and blood cancers.

## Materials and Methods

### Data Acquisition

HSC sequences and phylogenies for 10 healthy people (age range infant to 81) were retrieved from Mitchell et al. (2022) and for 15 individuals with MPN (age range 20 to 83) from Williams et al. (2022). An alignment was not available for one healthy individual (KX007), but the phylogeny was so it was used. HSC sequences were composed of genomic positions with SNAs. For MPN patients, the first sampling event for each individual was considered in order to eliminate bias stemming from cancer treatments. Two healthy infants were excluded from rate estimation and model fitting because they were still experiencing rapid HSC diversification among founder lineages and thus unrepresentative of the stable adult phase of HSC growth that we aimed to model. The incidence of MPN by age was obtained from Hultcrantz et al. (2020).

### Genetic Distance Estimation

We produced counts of sequence differences using MEGA to calculate pairwise GDs between HSC sequences (Tamura et al. 2021). In all these comparisons, positions with missing data were ignored (pairwise deletion option). This number was divided by 2 to generate per-sequence GD estimates. To determine the peak of the GD distribution, we generated a histogram with 300 GD bins and then identified the bin containing the highest point in the resulting GD distribution. We also counted the number of SNAs in an HSC sequence by comparing it with the respective germline genomes.

### Constant Versus Relaxed HSC Molecular Clocks

We used a likelihood ratio test (Wilks 1938; Glover and Dixon 2004) to compare fits between linear and polynomial regression models for GDs and SNAs accumulated in the HSCs of 7 adults. The polynomial models fit the data significantly better (*P* < 0.01) with the following models obtained for GDs and SNAs:


$$\begin{array}{rl} \mathrm{GDs} & =0.06\times \mathrm{ag}e^{2}+11.90\times \mathrm{age};\\ \mathrm{SNAs} & =0.06\times \mathrm{ag}e^{2}+12.43\times \mathrm{age} \end{array}$$

### *α* and *β* Parameter Estimation and Normalization

To calculate Colless’ index of phylogeny imbalance (Colless 1982), we used apTreeshape (Bortolussi et al. 2006) in R (R Core Development Team 2020). We plotted a LTT plot for each phylogeny using the R package ape (Paradis and Schliep 2019) and identified the point of inflection where lineage diversification ceased, corresponding to the end of the embryonic phase of HSC diversification (Lee-Six et al. 2018; Mitchell et al. 2022). Because our metrics assess the number and branching patterns of phylogenetic tips, an ultrametric HSC phylogeny was only required to make the initial crop of LTT. No estimates of an absolute time scale were necessary, which avoids biases associated with molecular dating approaches that may not work well when the evolutionary rates converge throughout the tree.

We generated the ancestral (embryonic) tree by cropping the HSC phylogeny at this inflection point and estimated the ancestral estimates of *π* and *n*. For some HSC phylogenies, apTreeshape failed to provide any estimates because the embryonic phylogeny contained fewer than 4 ancestral HSC lineages. In this case, we assumed log(*π_a_*) = 0 such that imbalance was the smallest. Meanwhile, this approach will work even when the HSC tree contains only the subclonal lineages because they will be distinguishable due to a long stem branch connecting them to the germline reference.

For individuals who were sampled multiple times in the progression of their cancer, we take only the first sampling event as their adult phylogeny condition. This avoids the confounding effects of the different treatments these individuals underwent to eliminate cancerous HSCs in their blood, necessarily impacting their HSC phylogenies.

### Modeling HSC Phylogeny Age

Both *α* and *β* increase exponentially with age (Fig. 6). Therefore, we fitted nonlinear models for estimating age using *α* and *β* separately. The model parameters were optimized using a numerical Gauss–Newton algorithm, aiming to minimize the sum of squared differences between the observed and predicted age of healthy individuals. Next, we combined these 2 models using a meta-regression framework (Viechtbauer 2010) with a maximum likelihood approach (Hardy and Thompson 1996). This process predicted the age and standard error for each healthy individual by utilizing random-effects meta-analysis in the metafor package in R (Viechtbauer 2010). We validated the combined model, predicted the age, and estimated the prediction interval for each healthy individual using the LOO approach, in which individual ages were estimated using a model that excluded the individual of interest.

### Annotations of Driver Mutations

Driver mutation counts per individual were obtained from the original studies, i.e. Mitchell et al. (2022) for healthy individuals and Williams et al. (2022) for individuals with MPN. Overall, we obtained information on the following proteins that contained driver mutations: ASXL1, CBL, DMNT3A, JAK2, KRAS, PPM1D, SF3B1, TET2, and TP53 in healthy people and CBL, DMNT3A, JAK2, PPM1D, TET2, 1q+, 7p−, 7q−, 9pUPD, 9q−, and 9+ in the MPN patients.

### Mutational Signature Analysis

We annotated a clade of many cells separated from the other cells with a long branch in a phylogeny. We identified singletons in cell genomes from the selected clade and inferred mutational signatures using Signal (Degasperi et al. 2020).

## Data Availability

Source code and sequence data are deposited in the Code Ocean online resource.

## References

[msad279-B1] Ahmad H, Jahn N, Jaiswal S. Clonal hematopoiesis and its impact on human health. Annu Rev Med. 2023:74(1):249–260. 10.1146/annurev-med-042921-112347. 36450282

[msad279-B2] Alexandrov LB, Kim J, Haradhvala NJ, Huang MN, Tian Ng AW, Wu Y, Boot A, Covington KR, Gordenin DA, Bergstrom EN, et al The repertoire of mutational signatures in human cancer. Nature. 2020:578(7793):94–101. 10.1038/s41586-020-1943-3. 32025018 PMC7054213

[msad279-B3] Ayachi S, Buscarlet M, Busque L. 60 Years of clonal hematopoiesis research: from X-chromosome inactivation studies to the identification of driver mutations. Exp Hematol. 2020:83:2–11. 10.1016/j.exphem.2020.01.008. 32001340

[msad279-B4] Bailey MH, Tokheim C, Porta-Pardo E, Sengupta S, Bertrand D, Weerasinghe A, Colaprico A, Wendl MC, Kim J, Reardon B, et al Comprehensive characterization of cancer driver genes and mutations. Cell. 2018:173(2):371–385.e18. 10.1016/j.cell.2018.02.060. 29625053 PMC6029450

[msad279-B5] Bell CG, Lowe R, Adams PD, Baccarelli AA, Beck S, Bell JT, Christensen BC, Gladyshev VN, Heijmans BT, Horvath S, et al DNA methylation aging clocks: challenges and recommendations. Genome Biol. 2019:20(1):249. 10.1186/s13059-019-1824-y. 31767039 PMC6876109

[msad279-B6] Beyene HB, Olshansky G, Smith AA T, Giles C, Huynh K, Cinel M, Mellett NA, Cadby G, Hung J, Hui J, et al High-coverage plasma lipidomics reveals novel sex-specific lipidomic fingerprints of age and BMI: evidence from two large population cohort studies. PLoS Biol. 2020:18(9):e3000870. 10.1371/journal.pbio.3000870. 32986697 PMC7544135

[msad279-B7] Bick AG, Weinstock JS, Nandakumar SK, Fulco CP, Bao EL, Zekavat SM, Szeto MD, Liao X, Leventhal MJ, Nasser J, et al Inherited causes of clonal haematopoiesis in 97,691 whole genomes. Nature. 2020:586(7831):763–768. 10.1038/s41586-020-2819-2. 33057201 PMC7944936

[msad279-B8] Borelli V . Glycomics as an innovative technology to identify biomarkers of aging [dissertation thesis]. Alma Mater Studiorum Università di Bologna, Italy. Dottorato di ricerca in Oncologia e patologia sperimentale, 26 Ciclo. 2014. 10.6092/unibo/amsdottorato/6312.

[msad279-B9] Bortolussi N, Durand E, Blum M, François O. apTreeshape: statistical analysis of phylogenetic tree shape. Bioinformatics. 2006:22(3):363–364. 10.1093/bioinformatics/bti798. 16322049

[msad279-B10] Brown AL, Li M, Goncearenco A, Panchenko AR. Finding driver mutations in cancer: elucidating the role of background mutational processes. PLoS Comput Biol. 2019:15(4):e1006981. 10.1371/journal.pcbi.1006981. 31034466 PMC6508748

[msad279-B11] Buscarlet M, Provost S, Zada YF, Barhdadi A, Bourgoin V, Lépine G, Mollica L, Szuber N, Dubé MP, Busque L. DNMT3A and TET2 dominate clonal hematopoiesis and demonstrate benign phenotypes and different genetic predispositions. Blood. 2017:130(6):753–762. 10.1182/blood-2017-04-777029. 28655780

[msad279-B12] Colless DH . Review: phylogenetics: the theory and practice of phylogenetic systematics by E. O. Wiley. Syst Zool. 1982:31(1):100–110. 10.2307/2413420.

[msad279-B13] Csordas A, Sipos B, Kurucova T, Volfova A, Zamola F, Tichy B, Hicks DG. Cell tree rings: the shape of somatic evolution as a human aging timer. bioRxiv 520419. 10.1101/2022.12.14.520419, 16 December 2022, preprint: not peer reviewed. PMC11009167 38172489

[msad279-B14] Dabrowski JK, Yang EJ, Crofts SJ, Hillary RF, Simpson DJ, Mccartney DL, Marioni RE, Latorre-Crespo E, Chandra T. Probabilistic inference of epigenetic age acceleration from cellular dynamics. bioRxiv 530570. 10.1101/2023.03.01.530570, 2 March 2023, preprint: not peer reviewed. PMC11485233 39313745

[msad279-B15] Degasperi A, Amarante TD, Czarnecki J, Shooter S, Zou X, Glodzik D, Morganella S, Nanda AS, Badja C, Koh G, et al A practical framework and online tool for mutational signature analyses show inter-tissue variation and driver dependencies. Nat Cancer. 2020:1(2):249–263. 10.1038/s43018-020-0027-5. 32118208 PMC7048622

[msad279-B16] Degasperi A, Zou X, Amarante TD, Martinez-Martinez A, Koh GCC, Dias JML, Heskin L, Chmelova L, Rinaldi G, Wang VYW, et al Substitution mutational signatures in whole-genome-sequenced cancers in the UK population. Science. 2022:376(6591):science.abl9283. 10.1126/science.abl9283. 35949260 PMC7613262

[msad279-B17] Dietlein F, Weghorn D, Taylor-Weiner A, Richters A, Reardon B, Liu D, Lander ES, Van Allen EM, Sunyaev SR. Identification of cancer driver genes based on nucleotide context. Nat Genet. 2020:52(2):208–218. 10.1038/s41588-019-0572-y. 32015527 PMC7031046

[msad279-B18] Ding M, Rexrode KM. A review of lipidomics of cardiovascular disease highlights the importance of isolating lipoproteins. Metabolites. 2020:10(4):163. 10.3390/metabo10040163. 32340170 PMC7240942

[msad279-B19] Fabre MA, de Almeida JG, Fiorillo E, Mitchell E, Damaskou A, Rak J, Orrù V, Marongiu M, Chapman MS, Vijayabaskar MS, et al The longitudinal dynamics and natural history of clonal hematopoiesis. Nature. 2022:606(7913):335–342. 10.1038/s41586-022-04785-z. 35650444 PMC9177423

[msad279-B20] Fancello L, Gandini S, Pelicci PG, Mazzarella L. Tumor mutational burden quantification from targeted gene panels: major advancements and challenges. J Immunother Cancer. 2019:7(1):183. 10.1186/s40425-019-0647-4. 31307554 PMC6631597

[msad279-B21] Franceschi C, Garagnani P, Parini P, Giuliani C, Santoro A. Inflammaging: a new immune-metabolic viewpoint for age-related diseases. Nat Rev Endocrinol. 2018:14(10):576–590. 10.1038/s41574-018-0059-4. 30046148

[msad279-B22] Galkin F, Mamoshina P, Kochetov K, Sidorenko D, Zhavoronkov A. DeepMAge: a methylation aging clock developed with deep learning. Aging Dis. 2021:12(5):1252–1262. 10.14336/AD.2020.1202. 34341706 PMC8279523

[msad279-B23] Gaunitz S, Tjernberg LO, Schedin-Weiss S. What can N-glycomics and N-glycoproteomics of cerebrospinal fluid tell us about Alzheimer disease? Biomolecules. 2021:11(6):858. 10.3390/biom11060858. 34207636 PMC8226827

[msad279-B24] Glover S, Dixon P. Likelihood ratios: a simple and flexible statistic for empirical psychologists. Psychon Bull Rev. 2004:11(5):791–806. 10.3758/BF03196706. 15732688

[msad279-B25] Goldman EA, Spellman PT, Agarwal A. Defining clonal hematopoiesis of indeterminate potential: evolutionary dynamics and detection under aging and inflammation. Cold Spring Harb Mol Case Stud. 2023:9(2):a006251. 10.1101/mcs.a006251. 36889927 PMC10240836

[msad279-B26] Groarke EM, Young NS. Aging and hematopoiesis. Clin Geriatr Med. 2019:35(3):285–293. 10.1016/j.cger.2019.03.001. 31230730 PMC8131033

[msad279-B27] Hannum G, Guinney J, Zhao L, Zhang L, Hughes G, Sadda S, Klotzle B, Bibikova M, Fan JB, Gao Y, et al Genome-wide methylation profiles reveal quantitative views of human aging rates. Mol Cell. 2013:49(2):359–367. 10.1016/j.molcel.2012.10.016. 23177740 PMC3780611

[msad279-B28] Hardy RJ, Thompson SG. A likelihood approach to meta-analysis with random effects. Stat Med. 1996:15(6):619–629. 10.1002/(sici)1097-0258(19960330)15:6<619::aid-sim188>3.0.co;2-a. 8731004

[msad279-B29] Horvath S . DNA methylation age of human tissues and cell types. Genome Biol. 2013:16(1):96. 10.1186/s13059-015-0649-6. PMC4427927 25968125

[msad279-B30] Horvath S, Ritz BR. Increased epigenetic age and granulocyte counts in the blood of Parkinson's disease patients. Aging. 2015:7(12):1130–1142. 10.18632/aging.100859. 26655927 PMC4712337

[msad279-B31] Hultcrantz M, Ravn Landtblom A, Andréasson B, Samuelsson J, Dickman PW, Kristinsson SY, Björkholm M, Andersson TM. Incidence of myeloproliferative neoplasms—trends by subgroup and age in a population-based study in Sweden. J Intern Med. 2020:287(4):448–454. 10.1111/joim.13019. 31927786 PMC7598815

[msad279-B32] Jaiswal S, Ebert BL. Clonal hematopoiesis in human aging and disease. Science. 2019:366(6465):eaan4673. 10.1126/science.aan4673. 31672865 PMC8050831

[msad279-B33] Jaiswal S, Natarajan P, Silver AJ, Gibson CJ, Bick AG, Shvartz E, McConkey M, Gupta N, Gabriel S, Ardissino D, et al Clonal hematopoiesis and risk of atherosclerotic cardiovascular disease. N Engl J Med. 2017:377(2):111–121. 10.1056/NEJMoa1701719. 28636844 PMC6717509

[msad279-B34] Krištić J, Vučković F, Menni C, Klarić L, Keser T, Beceheli I, Pučić-Baković M, Novokmet M, Mangino M, Thaqi K, et al Glycans are a novel biomarker of chronological and biological ages. J Gerontol A Biol Sci Med Sci. 2014:69(7):779–789. 10.1093/gerona/glt190. 24325898 PMC4049143

[msad279-B35] Lee-Six H, Kent DG. Tracking hematopoietic stem cells and their progeny using whole-genome sequencing. Exp Hematol. 2020:83:12–24. 10.1016/j.exphem.2020.01.004. 32007478 PMC7118367

[msad279-B36] Lee-Six H, Øbro NF, Shepherd MS, Grossmann S, Dawson K, Belmonte M, Osborne RJ, Huntly BJP, Martincorena I, Anderson E, et al Population dynamics of normal human blood inferred from somatic mutations. Nature. 2018:561(7724):473–478. 10.1038/s41586-018-0497-0. 30185910 PMC6163040

[msad279-B37] Lu AT, Binder AM, Zhang J, Yan Q, Reiner AP, Cox SR, Corley J, Harris SE, Kuo PL, Moore AZ, et al DNA methylation GrimAge version 2. Aging. 2022:14(23):9484–9549. 10.18632/aging.204434. 36516495 PMC9792204

[msad279-B38] Lu AT, Quach A, Wilson JG, Reiner AP, Aviv A, Raj K, Hou L, Baccarelli AA, Li Y, Stewart JD, et al DNA methylation GrimAge strongly predicts lifespan and healthspan. Aging. 2019:11(2):303–327. 10.18632/aging.101684. 30669119 PMC6366976

[msad279-B39] Mikkola HKA, Orkin SH. The journey of developing hematopoietic stem cells. Development. 2006:133(19):3733–3744. 10.1242/dev.02568. 16968814

[msad279-B40] Mitchell SR, Gopakumar J, Jaiswal S. Insights into clonal hematopoiesis and its relation to cancer risk. Curr Opin Genet Dev. 2021:66:63–69. 10.1016/j.gde.2020.12.004. 33422951 PMC8388615

[msad279-B41] Mitchell E, Spencer Chapman M, Williams N, Dawson KJ, Mende N, Calderbank EF, Jung H, Mitchell T, Coorens THH, Spencer DH, et al Clonal dynamics of haematopoiesis across the human lifespan. Nature. 2022:606(7913):343–350. 10.1038/s41586-022-04786-y. 35650442 PMC9177428

[msad279-B42] Miyoshi E, Kamada Y, Suzuki T. Functional glycomics: application to medical science and hepatology. Hepatol Res. 2020:50(2):153–164. 10.1111/hepr.13459. 31750967

[msad279-B43] Nachun D, Lu AT, Bick AG, Natarajan P, Weinstock J, Szeto MD, Kathiresan S, Abecasis G, Taylor KD, Guo X, et al Clonal hematopoiesis associated with epigenetic aging and clinical outcomes. Aging Cell. 2021:20(6):e13366. 10.1111/acel.13366. 34050697 PMC8208788

[msad279-B44] Nussinov R, Jang H, Tsai CJ, Cheng F. Review: precision medicine and driver mutations: computational methods, functional assays and conformational principles for interpreting cancer drivers. PLoS Comput Biol. 2019:15(3):e1006658. 10.1371/journal.pcbi.1006658. 30921324 PMC6438456

[msad279-B45] Ogawa M . Differentiation and proliferation of hematopoietic stem cells. Blood. 1993:81(11):2844–2853. 10.1182/blood.V81.11.2844.2844. 8499622

[msad279-B46] Osorio FG, Rosendahl Huber A, Oka R, Verheul M, Patel SH, Hasaart K, de la Fonteijne L, Varela I, Camargo FD, van Boxtel R. Somatic mutations reveal lineage relationships and age-related mutagenesis in human hematopoiesis. Cell Rep. 2018:25(9):2308–2316.e4. 10.1016/j.celrep.2018.11.014. 30485801 PMC6289083

[msad279-B47] Paradis E, Schliep K. Ape 5.0: an environment for modern phylogenetics and evolutionary analyses in R. Bioinformatics. 2019:35(3):526–528. 10.1093/bioinformatics/bty633. 30016406

[msad279-B48] R Core Development Team . A language and environment for statistical computing. Vienna (Austria): R Foundation for Statistical Computing; 2020.

[msad279-B49] Seale K, Horvath S, Teschendorff A, Eynon N, Voisin S. Making sense of the ageing methylome. Nat Rev Genet. 2022:23(10):585–605. 10.1038/s41576-022-00477-6. 35501397

[msad279-B50] Sha D, Jin Z, Budczies J, Kluck K, Stenzinger A, Sinicrope FA. Tumor mutational burden as a predictive biomarker in solid tumors. Cancer Discov. 2020:10(12):1808–1825. 10.1158/2159-8290.CD-20-0522. 33139244 PMC7710563

[msad279-B51] Simpson DJ, Chandra T. Epigenetic age prediction. Aging Cell. 2021:20(9):e13452. 10.1111/acel.13452. 34415665 PMC8441394

[msad279-B52] Singh I, Singh A. Clonal hematopoiesis of indeterminate potential: current understanding and future directions. Curr Oncol Rep. 2023:25(6):539–547. 10.1007/s11912-023-01382-9. 36928826

[msad279-B53] Slade E, Irvin MR, Xie K, Arnett DK, Claas SA, Kind T, Fardo DW, Graf GA. Age and sex are associated with the plasma lipidome: findings from the GOLDN study. Lipids Health Dis. 2021:20(1):30. 10.1186/s12944-021-01456-2. 33812378 PMC8019182

[msad279-B54] Tamura K, Stecher G, Kumar S. MEGA11: molecular evolutionary genetics analysis version 11. Mol Biol Evol. 2021:38(7):3022–3027. 10.1093/molbev/msab120. 33892491 PMC8233496

[msad279-B55] Van Egeren D, Escabi J, Nguyen M, Liu S, Reilly CR, Patel S, Kamaz B, Kalyva M, DeAngelo DJ, Galinsky I, et al Reconstructing the lineage histories and differentiation trajectories of individual cancer cells in myeloproliferative neoplasms. Cell Stem Cell. 2021:28(3):514–523.e9. 10.1016/j.stem.2021.02.001. 33621486 PMC7939520

[msad279-B56] Viechtbauer W . Conducting meta-analyses in *R* with the **metafor** package. J Stat Softw. 2010:36:1–48. 10.18637/jss.v036.i03.

[msad279-B57] Wang R, Li B, Lam SM, Shui G. Integration of lipidomics and metabolomics for in-depth understanding of cellular mechanism and disease progression. J Genet Genomics. 2020:47(2):69–83. 10.1016/j.jgg.2019.11.009. 32178981

[msad279-B58] Weidner CI, Lin Q, Koch CM, Eisele L, Beier F, Ziegler P, Bauerschlag DO, Jöckel KH, Erbel R, Mühleisen TW, et al Aging of blood can be tracked by DNA methylation changes at just three CpG sites. Genome Biol. 2014:15(2):R24. 10.1186/gb-2014-15-2-r24. 24490752 PMC4053864

[msad279-B59] Whittaker RH . Evolution and measurement of species diversity. Taxon. 1972:21(2-3):213–251. 10.2307/1218190.

[msad279-B60] Wilks SS . The large-sample distribution of the likelihood ratio for testing composite hypotheses. Ann Math Stat. 1938:9(1):60–62. 10.1214/aoms/1177732360.

[msad279-B61] Williams N, Lee J, Mitchell E, Moore L, Baxter EJ, Hewinson J, Dawson KJ, Menzies A, Godfrey AL, Green AR, et al Life histories of myeloproliferative neoplasms inferred from phylogenies. Nature. 2022:602(7895):162–168. 10.1038/s41586-021-04312-6. 35058638

[msad279-B62] Wong WJ, Emdin C, Bick AG, Zekavat SM, Niroula A, Pirruccello JP, Dichtel L, Griffin G, Uddin MM, Gibson CJ, et al Clonal haematopoiesis and risk of chronic liver disease. Nature. 2023:616(7958):747–754. 10.1038/s41586-023-05857-4. 37046084 PMC10405350

[msad279-B63] Younes IE, Syler L, Hamed A. Review of clonal hematopoiesis, subtypes and its role in neoplasia and different morbidities. Leuk Res. 2023:130:107307. 10.1016/j.leukres.2023.107307. 37186988

[msad279-B64] Zhu T, Gao Y, Wang J, Li X, Shang S, Wang Y, Guo S, Zhou H, Liu H, Sun D, et al CancerClock: a DNA methylation age predictor to identify and characterize aging clock in pan-cancer. Front Bioeng Biotechnol. 2019:7:388. 10.3389/fbioe.2019.00388. 31867319 PMC6905170

